# Assessment of anorexia nervosa according to the DSM-5 alternative personality model using the SCID-5-AMPD diagnostic interview system

**DOI:** 10.1192/j.eurpsy.2024.180

**Published:** 2024-08-27

**Authors:** J. Bognár, D. B. Pólya, G. Purebl, J. Réthelyi, K. Bai-Nagy, S. Hamvas, J. Biliczki

**Affiliations:** ^1^Department of Pediatrics; ^2^Semmelweis University, Budapest, Hungary; ^3^Institute of Behavioural Sciences; ^4^Department of Psychiatry and Psychotherapy, Semmelweis University, Budapest, Hungary

## Abstract

**Introduction:**

Anorexia nervosa (AN) is a chronic disease that significantly impairs the quality of life, with a low (less than 50%) remission rate, the incidence of which is increasing and it appears at younger and younger ages.

**Objectives:**

Our aim is to facilitate effective and targeted therapy for anorexia nervosa by identifying personality traits and endophenotypes that aid diagnosis and identification of psychotherapeutic targets.

**Methods:**

AN patients aged 18-45 years (N=14 female patients in the current study) completed online questionnaires on personality traits (PID-5), eating disorder (EDI-1), emotion regulation style, mentalization (MZQ), dissociation (DIS-Q), current emotional and mood state (SCL-90, PHQ-9), and past traumatic events (CTQ) after MINI and SCID-5-AMPD interview. Results were compared with a matched healthy control sample.

**Results:**

Apart from AN, the most common comorbidity was depressive episode, and anxiety disorders were also present. In the SCID-5-AMPD interview, high scores were obtained for several domains describing personality dysfunction in the AN group. Among self-report questionnaires, we observed significant differences in scores on the SCL-90-R, and no difference in CTQ in the AN patients compared to healthy controls.

**Conclusions:**

Based on the results of our study, the AN group showed more severe personality trauma, especially in the functional domains of identity and intimacy, and more psychological distress. The above may help to identify personalised psychotherapeutic treatment targets in AN patients, which may significantly improve effectiveness and reduce the time spent in therapy.

**Disclosure of Interest:**

None Declared

